# Low Serum Lysosomal Acid Lipase Activity Correlates with Advanced Liver Disease

**DOI:** 10.3390/ijms17030312

**Published:** 2016-02-27

**Authors:** Eyal Shteyer, Rivka Villenchik, Mahmud Mahamid, Nidaa Nator, Rifaat Safadi

**Affiliations:** 1 The Liver Unit, Gastroenterology Institute, Hadassah Medical Center, Hadassah Medical School, The Hebrew University, Jerusalem 9112001, Israel; eshteyer@hotmail.com (R.V.); nidaa@hadassah.org.il (N.N.); safadi@hadassah.org.il (R.S.); 2 Pediatric Gastroenterology Institute, Shaare Zedek Medical Center, Hadassah Medical School, The Hebrew University, Jerusalem 9103102, Israel; 3 Liver Unit, Gastroenterology Institute, Shaare Zedek Medical Center, Hadassah Medical School, The Hebrew University, Jerusalem 9112001, Israel; mmahamid@szmc.org.il; 4 Liver Unit, Holy Family Hospital; Safed Medical School, Bar Ilan University, Nazareth 1641110, Israel

**Keywords:** lysosomal acid lipase, cholesteryl ester storage disease, non-alcoholic liver disease, non-alcoholic steatohepatitis, cirrhosis

## Abstract

Fatty liver has become the most common liver disorder and is recognized as a major health burden in the Western world. The causes for disease progression are not fully elucidated but lysosomal impairment is suggested. Here we evaluate a possible role for lysosomal acid lipase (LAL) activity in liver disease. To study LAL levels in patients with microvesicular, idiopathic cirrhosis and nonalcoholic fatty liver disease (NAFLD). Medical records of patients with microvesicular steatosis, cryptogenic cirrhosis and NAFLD, diagnosed on the basis of liver biopsies, were included in the study. Measured serum LAL activity was correlated to clinical, laboratory, imaging and pathological data. No patient exhibited LAL activity compatible with genetic LAL deficiency. However, serum LAL activity inversely predicted liver disease severity. A LAL level of 0.5 was the most sensitive for detecting both histologic and noninvasive markers for disease severity, including lower white blood cell count and calcium, and elevated γ-glutamyltransferase, creatinine, glucose, glycated hemoglobin, uric acid and coagulation function. Serum LAL activity <0.5 indicates severe liver injury in patients with fatty liver and cirrhosis. Further studies should define the direct role of LAL in liver disease severity and consider the possibility of replacement therapy.

## 1. Introduction

Fatty liver has become the most common liver disorder [1] and is recognized as a major health burden in the Western world. The spectrum of histological abnormalities includes simple steatosis (steatosis without other liver injuries) and nonalcoholic steatohepatitis in its more extreme forms [2]. Over 30% of adults in developed countries suffer from hepatic fat accumulation [3]. Among these patients, 60% are diabetic, obese or morbidly obese [3,4,5].

The earliest stage of nonalcoholic fatty liver disease (NAFLD) consists of hepatic steatosis or lipid deposition in the cytoplasm of hepatocytes [6,7]. Hepatic steatosis may progress to the more aggressive necro-inflammatory form of NAFLD, nonalcoholic steatohepatitis (NASH) [2]. NASH patients, as compared to those with steatosis, have a much greater risk for developing liver cirrhosis, a significant risk factor for development of hepatocellular carcinoma [7,8,9]. It is still unclear what leads to the progression from simple steatosis to advanced liver disease. In some cases hepatic steatosis is merely a marker for other diseases, such as microvesicular steatosis in metabolic diseases [10] and in viral hepatitis [11].

An emerging cause for fatty liver and hepatic dysfunction is lysosomal acid lipase deficiency (LAL-d). Pronounced LAL-d is a rare autosomal recessive storage disorder, leading to lysosomal accumulation of lipids, predominately cholesteryl esters and triglycerides in various tissues and cell types. In LAL-deficient hepatocytes increased levels of cholesterol lead to substantial increases in very low-density lipoprotein (VLDL)-cholesterol production and secretion, the normal way of exporting cholesterol from the liver. This in turn leads to enhanced low-density lipoprotein (LDL)-cholesterol secretion and thus may be an important enhancer of hypercholesterolemia in LAL-d [12]. LAL-d is classified as either Wolman disease (WD) or cholesteryl ester storage disease (CESD), both characterized by very low LAL activity [13,14,15]. CESD usually has a later onset than WD, and primarily affects the liver, with a wide spectrum of involvement ranging from early onset disease with severe cirrhosis to later onset of slowly progressive hepatic disease with survival into adulthood. Subsequently, complications of fatty liver disease with mixed hyperlipidemia lead to accelerated atherosclerosis, which dominates the clinical picture. Moreover, CESD patients exhibit many abnormalities that overlap with those in more common liver disorders such as nonalcoholic fatty liver disease (NAFLD), making the diagnosis of CESD much more challenging. Therefore, the importance of LAL-d in dyslipidemia and liver dysfunction was recently suggested for the NAFLD spectrum [9]. Furthermore, low LAL activity has been reported only in patients with NAFLD, underscoring the potential role of LAL in NAFLD [16].

The aim of the current study was to further evaluate LAL activity in patients with liver diseases that may be attributed to LAL-d: fatty liver with microvesicular steatosis, cryptogenic cirrhosis and NAFLD.

## 2. Results

### 2.1. Basic Characterization of the Study Population

Seventy-four patients diagnosed with cirrhosis according to the International Classification of Diseases 9 (ICD9) classification, and having an available liver biopsy were identified. Sixty-three were excluded due to clear etiology for their liver disease, thus not meeting the diagnostic criteria for cryptogenic cirrhosis. Two of the remaining patients underwent liver transplantation and five others declined to participate in the study. From the 15 patients with histology of microvesicular steatosis, two were excluded due to other overt etiology and four patients refused to participate in the trial. Nine NAFLD-patients with macrovesicular steatosis were also included. Altogether, the 22 patients in the study were analyzed as one group and as two groups, designated as higher-risk for LAL-d (13 patients, nine with microvesicular steatosis and four with cryptogenic cirrhosis) and lower-risk for LAL-d (nine patients with metabolic syndrome and NAFLD).

The mean age of all 22 patients participating in the study was 32.4 ± 23.3 (range 3.0–71.8) years, with similar distribution of males and females (Table 1). The ethnic origin of most participants was Arab and the rest were defined as Ashkenazi or Sephardi Jews. The age of the high-risk group was significantly lower (*p* = 0.001), while the rate of consanguinity and family history of fatty liver or cirrhosis were higher in this group (*p* > 0.05). As expected, systemic blood pressure, body mass index (BMI), and waist circumference were significantly higher in the low-risk group (*p* = 0.023–0.028, *p* = 0.006 and *p* = 0.006, respectively).

Differences between groups were found for several laboratory tests. Alkaline phosphatase serum levels were significantly higher in the high-risk group (198.5 ± 76 *vs.* 94 ± 33. *p* < 0.001); this may be attributed to the younger age of the patients in this group. In contrast, the low-risk group had significantly higher levels of urea (8.3 ± 2 *vs.* 11.9 ± 2.9 *p* < 0.006), uric acid (234.8 ± 50 *vs.* 347 ± 66, *p* < 0.006) and hematocrit (36.3 ± 5 *vs.* 41 ± 4, *p* < 0.03). A significant difference was also noted in white blood cell count (WBC), glycated hemoglobin (HbA1c) and thyroid-stimulating hormone (TSH). Abdominal imaging and liver histologic assessments showed higher fibrosis scorings in the high-risk group (*p* = 0.01). However, imaging signs of portal hypertension and NAS biopsy scores were similar (Table 2).

### 2.2. Lysosomal Acid Lipase (LAL) Activity

Mean LAL activity was 0.74 (median 0.8, ±0.28) nmol/punch/h, and was similar in both risk groups. Subsequently, the entire cohort was analyzed according to two LAL cutoffs: 0.5 and 0.6 nmol/punch/h. Characterization of the cohort according to the cutoffs revealed similar composition with respect to age, gender, origin, weight, MBI, waist circumference, smoking rate, consanguinity, family history (of fatty liver or cirrhosis) and blood pressure (Table 3).

Table 4 shows selected parameters that differed significantly when analyzed according to LAL cutoffs. Significant differences were found for WBC, platelets (PLT), International Normalized Ratio (INR), γ-glutamyltransferase (γGT), total protein, albumin, calcium, uric acid, creatinine, glucose and HbA1c. Other parameters that were analyzed but were not significantly different included hematological (hemoglobin, hematocrit (HCT)), biochemical (sodium, alanine aminotransferase (ALT), aspartate aminotransferase (AST), alkaline phosphatase (ALP), total bilirubin, direct bilirubin, phosphorous, urea, triglycerides, low-density lipoproteins (LDL), high-density lipoproteins (HDL) and total cholesterol), metabolic and inflammatory markers (TSH, vitamin D 25, ammonia, ferritin, C-reactive protein (CRP)), as well as α-fetoprotein (αFP). A threshold of LAL <0.5 was found to characterize six patients. All had marked macrosteatosis and hepatomegaly. LAL <0.5 identified eight severity markers of liver disease, including low calcium levels, a low WBC, high creatinine levels, high uric acid, high glucose and HbA1c, and high γGT and prolonged INR. The seven patients with a LAL threshold <0.6 were the six mentioned above (for the LAL <0.5 threshold) and a child from the high-risk group with severe microvesicular steatosis and liver fibrosis complicated by portal hypertension. LAL <0.6 identified seven additional markers, including lower serum calcium, total protein and platelets, and increased glucose, HbA1c, uric acid and INR.

Abdominal imaging and liver histologi c characterization were also analyzed according to the LAL cutoffs (Table 5). There were no significant differences between LAL-groups. However, in the ≥0.5 group the NAS score was significantly higher and the fibrosis score was marginally higher compared to the <0.5 group (*p* = 0.06) (Table 5). In conclusion, the LAL 0.5 threshold was the most sensitive for detecting both histologic and noninvasive markers for disease severity.

## 3. Discussion

Fatty liver disease is emerging as the leading liver disease with no current effective treatment. Although in most cases a metabolic syndrome is the cause of hepatic steatosis, other causes of fatty liver should also be considered. One of those diagnoses is lysosomal acid lipase deficiency (LAL-d), which is hopefully soon to be treatable with encouraging results from enzyme replacement therapy (Sebelipase Alfa, Kanuma^®^, New Haven, CT, USA). This was indeed our initial motivation for the current study. We aimed to assess LAL activity in patients with liver disease in order to provide suitable therapy. Thus, we measured levels of LAL in patients with cryptogenic cirrhosis, microvesicular steatosis and nonalcoholic fatty liver disease (NAFLD) related to a metabolic syndrome. Although no LAL-d was found, and no patient was eligible for enzyme replacement therapy, we did find that low LAL activity was associated with liver disease severity.

Our initial aim in the study was to compare patients with higher likelihood of genetically-low LAL activity (cryptogenic cirrhosis and microvesicular steatosis) to patients with NAFLD who we thought would be less likely to have low LAL activity. However, Baratta *et al.* [16] reported recently that patients with NAFLD have low LAL activity. As we could not find any statistical difference in LAL levels when we compared the two groups, we concluded that our study supports the study by Baratta *et al.* [16]. Subsequently, we analyzed our data according to two LAL levels. The analysis revealed significant differences that could be attributed to liver disease severity. A LAL threshold of 0.5 identified six patients with significantly higher histologic scorings and eight noninvasive markers (including low calcium levels and white blood cell count, and high creatinine, uric acid, glucose and HbA1c, and γGT levels and prolonged INR). A LAL threshold of 0.6 detected seven patients with seven markers (including low PLT count, calcium levels and total protein; prolonged INR; and high uric acid, glucose and HbA1c), but could not differentiate on the basis of histologic severity.

The blood work that was found to be different in patients with low LAL activity levels signifies indirect measures for liver disease severity. Low platelets and white blood counts serve as indirect markers for cirrhosis because of portal hypertension and hypersplenism. An elevated creatinine level, which is a marker of advanced liver disease and a strong predictor of survival in cirrhosis and [17] hepatorenal syndrome patients [18,19,20], was also observed in the lower-risk LAL group. With respect to insulin resistance, higher glucose and HbA1c levels were also observed for patients in the low LAL group and may signify more advanced fatty liver disease [21]. Interestingly, higher γGT levels were observed in the lower risk LAL group. This observation corresponds with the other disease severity markers, as γGT is regarded to be an independent predictive marker of morbidity and mortality in cardiovascular-related disorders, including coronary arterial disease, and congestive heart failure [17,22,23,24,25]. Higher uric acid levels may be a result of hypovolemia but also of advanced liver injury, accompanied by malnutrition and protein breakup, or a secondary renal injury [21]. Furthermore, when assessing the NAS score we found higher scores for NASH and fibrosis at low LAL levels. Taken together, all measures that were found to be different in the low LAL group signify hepatic and overall disease severity.

The association between low LAL activity and severity of liver injury merits further discussion. It may be considered that low LAL activity in patients with severe liver disease is merely a consequence of an overall decrease in viable hepatocytes that leads to lower protein production. On the other hand, various studies in animal models suggest that lower LAL activity may be part of the pathogenesis of fatty liver disease. The mechanism of lipid accumulation in hepatocytes is not completely elucidated but the role of lipases, including LAL is significant [26]. Autophagy is the key process in hepatic lipid metabolism and steatosis [27], and is the common pathway for the other liver diseases included in our study. Thus, other enzymes may be affected in our cohort. Nevertheless, the importance of measuring LAL activity lies in the potential for treatment with enzyme replacement therapy. Furthermore, the lysosomal-associated NK cells are crucial to prevent fibrosis progression in liver diseases [28,29] and LAL decrease uncovers an additional possible mechanism.

The major limitation of the study is the number of patients and the age range. Despite these limitations we still observed significant differences between the groups of patients with lower and higher LAL activity. It is hard to draw clear conclusions from these observation but they may set a basis for further studies to elucidate the role of LAL in each group of patients within a larger cohort.

## 4. Materials and Methods

### 4.1. Study Design

This study was conducted in the Liver Unit, Hadassah Medical Center, Jerusalem, Israel. The local ethics committee of Hadassah Medical Center approved the study (application 920120061, 24/05/2012) and written informed consent was obtained from all the participants or legal guardians in cases of minors. Patients aged 1–75 years who underwent liver biopsy during the years 2006–2012 were screened for the diagnosis of cryptogenic cirrhosis (according to ICD9 registration), microvesicular steatosis (according to liver pathology reports) and NAFLD with macrovesicular steatosis.

Exclusion criteria included daily alcohol intake >10 g/day, exposure to any other hepatotoxic agents, or evidence of other liver disease. Therefore, patients were excluded with the presence of serum hepatitis B surface antigen (HBsAg), hepatitis C viral (HCV) antibodies, HCV RNA, positive autoimmune serology, evidence for hemochromatosis, Wilson’s disease (low ceruloplasmin serum levels and high liver tissue copper content) or α-1-antitrypsin disease (low α-1-antitrypsin levels with suggestive biopsy). Abdominal ultrasound was performed to exclude masses, obstruction of bile or blood vessels, but also provided features of liver steatosis and cirrhosis.

### 4.2. Study Groups

The cohort of patients was analyzed both as a whole group and as two groups: one consisting of patients with cryptogenic cirrhosis or microvesicular steatosis, and a second consisting of patients with NAFLD and macrovesicular steatosis.

### 4.3. Clinical Characterizations

Body mass index (BMI), blood pressure, waist circumference, concomitant diseases and medications were recorded at the time of LAL evaluation. Any results of abdominal imaging (Abdominal Ultrasound, Computerized Tomography and Magnetic Resonance) were documented, focusing on fatty liver appearance, hepatomegaly and splenomegaly and hepatic fibrosis (irregular hepatic appearance).

### 4.4. NAFLD Activity Score (NAS)

This score represented the sum of scores for steatosis, lobular inflammation, and ballooning, ranging from 0 to 8 according to Kleiner *et al.* [30]. Subjects with a NAS activity score of 0–2 were considered as having NAFLD. Biopsies with an activity score of 3 or more were considered as NASH. Fibrosis was ranked as follows: 0-none, 1-perisinusoidal or periportal, 2-perisinusoidal and periportal, 3-bridging fibrosis, 4-cirrhosis.

### 4.5. LAL Activity in Dried Blood Spots (DBS)

The test was performed as described previously by Hamilton *et al.* [31]. DBS values of 0.37–2.30 nmol/punch/h were interpreted as normal, 0.15–0.40 nmol/punch/h as carriers and <0.03 nmol/punch/h as CESD patients.

### 4.6. Statistical Analysis

All clinical, laboratory, imaging and pathological parameters were compared between the two groups using the *t*-test and the nonparametric Mann-Whitney *U* test. Categorical parameters were compared using Fisher’s exact test. All statistical tests were bilateral and a *p*-value of 5% or less was considered statistically significant.

## 5. Conclusions

In the current study we found that LAL activity correlates with hepatic steatosis and dysfunction. Our findings suggest a possible role for LAL in the pathogenesis of liver dysfunction and future studies may assist in finding subseta of patients who will benefit from enzyme replacement therapy. As our cohort is small, further larger groups should be studied in order to substantiate our findings.

## Figures and Tables

**Table 1:** Baseline characteristics of participants.

Parmeters	Discriptors	High Risk *n* = 13	Low Risk *n* = 9	Total *n* = 22	*p*
Age, years	Mean ± SD	17.2 ± 12.3	54.3 ± 17.1	32.4 ± 23.3	0.0001
	Median	14.2	59.2	24.9	-
	Range	3.0–39.9	21.7–71.8	3.0–71.8	-
Gender, Male, %	-	61.5	44.4	54.5	0.666
Origin, %	Ashkenazi Jew	15.4	22.2	18.2	1.000
	Sephardi Jew	7.7	11.1	9.1	-
	Arab	76.9	66.7	72.7	-
Consanguinity, %	-	58.3	22.2	42.9	0.184
Familial Fatty liver, %	-	58.3	12.5	40	0.070
Familial Cirrhosis, %	-	33.3	0	21.1	0.245
Smoking, %	-	15.4	33.3	22.7	0.609
SBP, mmHg	Mean ± SD	116.9 ± 10.3	128.2 ± 10.3	121.8 ± 11.6	0.028
	Median	117.0	131.0	125.0	-
DBP, mmHg	Mean ± SD	66.1 ± 14.8	77.9 ± 6.1	71.1 ± 13.1	0.023
	Median	69.5	79.0	74.0	-
BMI, kg/m^2^	Mean ± SD	22.1 ± 6.8	33.4 ± 8.5	28.0 ± 89.5	0.006
	Median	19.95	30.1	26.2	-
Waist C., m	Mean ± SD	0.79 ± 0.11	1.07 ± 0.16	0.98 ± 0.2	0.006
	Median	0.80	1.01	0.95	-

SBP = Systolic blood pressure; DBP = Diastolic blood pressure; BMI = Body mass index; Waist C. = Waist circumference; SD = Standard deviation; m = meters; *n* = number of patients. *p* Value calculated by: Fisher’s Exact Test, Exact Significance (2-sided); Mann-Whitney Test, Exact Significance (2*(1-tailed Sig.)).

**Table 2:** Imaging and histologic characterization of participants.

Total (*n* = 22)	High Risk Study Group (*n* = 13)	Low Risk Control Group (*n* = 9)	Total (*n* = 22)	*p* Value
Fatty liver, Imaging test, *n*	4 (31%)	9 (100%)	13 (59%)	0.002
Hepatomegaly, Imaging test, *n*	6 (46%)	2 (22%)	8 (36%)	0.380
Splenomegaly, Imaging test, *n*	6 (46%)	3 (33%)	9 (41%)	0.674
Hepatic Fibrosis, Imaging test, *n*	3 (23%)	1 (11%)	4 (18%)	0.616
Portal Hypertension, Imaging test, *n*	3 (23%)	1 (11%)	4 (18%)	0.616
Macrovesicular steatosis, Liver pathology, *n*	7 (54%)	2 (22%)	9 (41%)	0.620
Microvesicular steatosis, Liver pathology, *n*	7 (54%)	0	7 (32%)	0.044
Liver fibrosis score, mean ± SD	2.4 ± 1.1	1 ± 1.3	1.9 ± 1.3	0.01
NAS scoring, Liver pathology	2.8 ± 2	2.2 ± 2.2	2.6 ± 2	1.000

Imaging test = Ultrasound (US), Computed tomography (CT) or Magnetic resonance imaging (MRI); *p* value calculated by Fisher’s Exact Test, Exact Significance (2-sided).

**Table 3:** Baseline characteristics of participants according to LAL cutoffs.

Parmeters	Discriptors	LAL 0.5 Cutoff			LAL 0.6 Cutoff		
		<0.5	≥0.5	*p*	<0.6	≥0.6	*p*
		(*n* = 6)	(*n* = 16)		(*n* = 7)	(*n* = 15)	
Age, years	Mean ± SD	46.3 ± 18.4	27.2 ± 23.3	0.08	40.7 ± 22.4	28.5 ± 23.5	0.26
	Median	52.5	18.6		47.4	21.7	
Males, *n*		2	10	0.34	3	9	0.65
Jew, *n*	Ashkenazi	2	2	0.57	2	2	0.60
Jew, *n*	Sephardi	0	2		0	2	
Arab, *n*	Palestinian	4	12		5	11	
Consanguinity , *n*		2	7	1.00	3	6	1.00
Familial Fatty liver, *n*		2	6	1.00	2	6	1.00
Familial Cirrhosis, *n*		0	4	1.00	0	4	0.53
Smoking, *n*		1	4	1.00	1	4	1.00
SBP, mmHg	Mean ± SD	122.17 ± 9.95	121.60 ± 12.52	0.97	121.14 ± 9.48	122.07 ± 12.85	0.69
	Median	122.50	126.00		120.00	126.00	
DBP, mmHg	Mean ± SD	79.17 ± 8.59	67.93 ± 13.39	0.09	75.86 ± 11.75	68.79 ± 13.47	0.29
	Median	77.50	70.00		74.00	71.50	
BMI, kg/m^2^	Mean ± SD	33.89 ± 12.77	25.66 ± 7.07	0.19	33.89 ± 12.77	25.66 ± 7.07	0.19
	Median	36.33	24.96		36.33	24.96	
Waist C., m	Mean ± SD	1.16 ± 0.21	0.90 ± 0.13	0.08	1.16 ± 0.21	0.90 ± 0.13	0.08
	Median	1.19	0.94		1.19	0.94	

SBP = Systolic blood pressure; DBP = Diastolic blood pressure; BMI = Body mass index; SD = Standard deviation; m = meters; *n* = number of patients; Waist C. = Waist Circumference. *p* Value calculated by: Fisher’s Exact Test, Exact Significance (2-sided); Mann-Whitney Test, Exact Significance (2*(1-tailed Sig.)).

**Table 4:** Laboratory results of participants, according to LAL cutoffs.

Parmeters	LAL Cutoff 0.5							LAL Cutoff 0.6						
	LAL < 0.5 *n* = 6			LAL ≥ 0.5 *n* = 16			*p*	LAL < 0.6 *n* = 7			LAL ≥ 0.6 *n* = 15			*p*
	Mean	Median	SD	Mean	Median	SD		Mean	Median	SD	Mean	Median	SD	
WBC	6.64	7.01	1.36	9.33	8.76	3.31	**0.029**	6.95	7.11	1.49	9.37	8.59	3.43	0.079
PLT(x1000)	193	207	96	292	273	110	0.095	194	200	87	298	284	111	**0.046**
INR	1.25	1.17	0.28	1.06	1.02	0.11	**0.046**	1.23	1.09	0.26	1.05	1.02	0.12	**0.022**
γGT	147.02	135.50	104.84	61.70	35.00	59.41	**0.036**	129.44	108.00	106.41	64.84	37.50	60.91	0.100
Total protein	66.80	67.00	10.83	75.86	76.00	6.14	0.070	66.67	67.00	9.69	76.62	76.00	5.66	**0.012**
Albumin	38.17	43.00	10.94	43.80	44.00	5.68	0.178	37.86	43.00	10.02	44.36	44.50	5.46	**0.056**
Calcium	2.34	2.38	0.19	2.53	2.46	0.12	**0.012**	2.34	2.38	0.19	2.53	2.46	0.12	**0.012**
Uric acid	382.25	380.26	64.44	269.43	280.00	64.18	**0.020**	382.25	380.26	64.44	269.43	280.00	64.18	**0.020**
Creatinine	64.14	62.03	20.71	44.78	45.00	17.75	**0.049**	60.12	60.00	21.69	45.37	45.76	18.22	0.142
Glucose	6.60	6.46	0.80	5.18	5.01	0.91	**0.005**	6.70	6.72	0.78	5.04	4.90	0.74	**<0.001**
HbA1c	6.12	6.10	0.55	5.51	5.50	0.18	**0.048**	6.12	6.10	0.55	5.51	5.50	0.18	**0.048**

*p* = *p* value, calculated by Mann-Whitney Test, Exact Significance [2*(1-tailed Sig)]. Significant values are in bold.

**Table 5:** Imaging and histologic characterization of participants according to LAL cutoffs.

LAL Cutoff	LAL 0.5			LAL 0.6		
	<0.5 (*n* = 6)	≥0.5 (*n* = 16)	*p*	<0.6 (*n* = 7)	≥0.6 (*n* = 15)	*p*
Fatty liver, Image, *n*	4	9	1.0	4	9	1.0
Hepatomegaly, Image, *n*	2	6	1.0	3	5	1.0
Splenomegaly, Image, *n*	4	5	0.18	5	4	0.07
Cirrhosis Liver, Image, *n*	1	3	1.0	1	3	1.0
PTH, Image, *n*	2	2	0.29	3	1	0.08
NAS score	2.1	3.7	0.03	3.3	2	0.1
Fibrosis score	1.75	3	0.06	1.8	2.75	0.1

*p* = *p*-value, calculated by Fisher’s Exact Test, Exact Significance (2-sided); NAS: Nonalcoholic steatohepatitis score, PTH = Portal Hypertension.
